# Association between routine cell salvage use for lower segment caesarean section and post-operative iron infusion and anemia

**DOI:** 10.1007/s00404-023-07082-w

**Published:** 2023-05-26

**Authors:** Tom P. Fox, Evelyn Timpani, Amanda Green, Anupam Parange, Romi Sinha, Thu-Lan Kelly, Nicolette A. Hodyl, Bernd Froessler

**Affiliations:** 1 Department of Anesthesia, Lyell McEwin Hospital, Elizabeth Vale, 5112 SA Australia; 2 Department of Obstetrics and Gynecology, Lyell McEwin Hospital, Elizabeth Vale, 5112 SA Australia; 3 Department of Health and Wellbeing, Blood Organ and Tissue Programs, Adelaide, 5000 SA Australia; 4 Clinical and Health Sciences, Quality Use of Medicines Pharmacy Research Centre, University of South Australia, Adelaide, 5000 SA Australia; 5 Hunter Medical Research Institute, New Lampton Heights, 2305 NSW Australia; 6 Discipline of Acute Care Medicine, Adelaide Medical School, The University of Adelaide, Adelaide, 5000 SA Australia

**Keywords:** Intraoperative Blood Cell Salvage, Anemia, Cesarean section, Patient blood management

## Abstract

**Purpose:**

Intraoperative cell salvage is central to Patient Blood Management including for lower segment caesarean section. Prior to April 2020, we initiated intraoperative cell salvage during caesarean section based on risk assessment for hemorrhage and patient factors. As the pandemic broadened, we mandated intraoperative cell salvage to prevent peri-partum anemia and potentially reduce blood product usage. We examined the association of routine intraoperative cell salvage on maternal outcomes.

**Methods:**

We conducted a single-center non-overlapping before-after study of obstetric patients undergoing lower segment caesarean section in the 2 months prior to a change in practice (‘usual care = selective intraoperative cell salvage’, *n* = 203) and the 2 months following (‘mandated intraoperative cell salvage’, *n* = 228). Recovered blood was processed when a minimal autologous reinfusion volume of 100 ml was expected. Post-operative iron infusion and length of stay were modelled using logistic or linear regression, using inverse probability weighting to account for confounding.

**Results:**

More emergency lower-segment caesarean sections occurred in the Usual Care group. Compared to the Usual Care group, post-operative hemoglobin was higher and anemia cases fewer in the Mandated intraoperative cell salvage group. Rates of post-partum iron infusion were significantly lower in the Mandated intraoperative cell salvage group (OR = 0.31, 95% CI = 0.12 to 0.80, *P* = 0.016). No difference was found for length of stay.

**Conclusion:**

Routine cell salvage provision during lower segment caesarean section was associated with a significant reduction in post-partum iron infusions, increased post-operative hemoglobin and reduced anemia prevalence.

## What does this study add to the clinical work

**Table:** 

Cell salvage provision during cesarean section is a valuable tool and reduces the incidence of post-partum anemia and the need for post-partum iron infusion. It may contribute to an avoidance or reduction of allogeneic red blood cell transfusion in the peri-partum setting.	

## Introduction

In March 2020, the World Health Organization (WHO) declared a pandemic status for the coronavirus disease 2019 (COVID-19). In addition to the unprecedented pressures on healthcare systems, COVID-19 has had a profound impact on the number of blood donations, leaving blood services severely challenged to maintain their inventory during the pandemic [1–3]. These challenges highlight the critical importance of Patient Blood Management (PBM) as an integral part of patient care. In a recent publication, a multinational and diverse group of authors issued a “Call to Action” urging all stakeholders and providers to implement practical and multimodal principles of PBM [4].

PBM is a patient-centered approach, applying an evidence-based bundle of care to preserve the patient’s own blood by managing anemia, minimizing iatrogenic blood loss, and harnessing tolerance to anemia to improve patient outcomes [5]. Intraoperative cell salvage (IOCS) is an integral and valuable component of PBM [5–7].

The benefits of IOCS have been known for some time [8]. Clinical adoption of IOCS in obstetrics for lower segment caesarean section (LSCS) has increased substantially, but despite this, the utilization of this technique varies considerably around the world [9–11]. At our institution, IOCS has been an established modality since 2013, as part of our hospital-wide PBM approach. Prior to April 2020, our institutional practice included initiation of IOCS during LSCS based on pre-operative anemia, patient risk assessment for intra-operative blood loss, post-partum hemorrhage and other individual patient factors.

In response to the Australian National Blood Authority’s call to adhere to PBM guidelines and practices during the pandemic, to preserve finite reserves of donated blood, [12, 13] members of the Departments of Anesthesia and Obstetrics at our institution discussed if modifications of our PBM practices were warranted to prevent the incidence and severity of peri-partum anemia. A decision was made to trial the mandated use of IOCS for all LSCS during the height of the first wave of the pandemic, to explore whether this practice could reduce the need for red blood cell (RBC) transfusion. We also aimed to examine the association of routine IOCS for LSCS on other indicators of optimal PBM, including the rate and volume of returned cell salvaged blood and the incidence and degree of post-partum anemia, as well as the impact on other maternal outcomes such as length of hospital stay.

## Methods

This study was approved by our Institutional Review Board (IRB), (CALHN Reference Number: 14994). Written informed consent was waived by the IRB. We conducted a single-center non-overlapping before-after study of all obstetric patients undergoing either elective or emergent lower segment caesarean section over a four-month period in early 2020. Exclusion criteria were caesarean section for stillbirth or patient refusal of the utilization of cell salvage, which was discussed while obtaining anesthesia consent. This study adheres to The Strengthening the Reporting of Observational Studies in Epidemiology (STROBE) Statement [14].

Data from selective intra-operative cell salvage practice (06 February 2020–05 April 2020) and the subsequent two months (06 April 2020–05 June 2020) with the use of routine intra-operative shed blood collection was collected from electronic and paper records, after the completion of the study period. Participants were identified via electronic hospital records, using the search terms “LSCS” and “Caesarean” between the above dates. Anemia was defined as hemoglobin (Hb) < 110 g/L during pregnancy and Hb < 100 g/L post-partum [13]. The risk of bias was low, as for both groups, the recovered blood was processed when a substantial blood loss occurred, and the autologous RBC reinfusion volume was expected to reach 100 ml or more.

### Statistical analysis

Descriptive statistics summarized patient characteristics using counts and percentages for categorical data, means and standard deviations or medians and interquartile ranges for continuous data, as appropriate. To compare post-operative outcomes between the groups who have mandated IOCS or received usual care, *t*-tests for normally distributed continuous data, Kruskal–Wallis tests for skewed data, and chi-square tests for categorical data were used.

Post-partum iron infusion was modelled using logistic regression, and log-transformed length of stay with linear regression. Other outcomes, such as post-operative hemoglobin or anemia, were not modelled due to a large proportion of missing data. Differences between mandated IOCS and usual care were expressed as odds ratios for post-partum iron infusion and geometric mean ratios for length of stay, and 95% confidence intervals (CI). To account for potential confounding by differences in patients between time periods and types of care, a counterfactual approach with propensity modelling was used. The propensity model used mandated IOCS vs usual care as the outcome with covariates age, parity, gravida, ASA score, pre-operative Hb, operation type and anesthesia type. The propensity model was used to create trimmed, stabilized inverse probability weights (IPW). Standardized mean differences (SMD) were used to assess balance in covariates at baseline before and after weighting, where an SMD > 0.1 was considered imbalanced.

Finally, to investigate the possibility that the changes over time could have occurred regardless of mandated IOCS, an interrupted time series (ITS) analysis, adapted for short time periods, was performed using the R package ‘its.analysis’. Post-partum IV iron and length of stay were aggregated by week to create time series. A significant difference in the outcome between the two periods was tested, accounting for important covariates and autocorrelation in the outcome over time. The ITS model for a number of post-operative iron infusions per week used pre-operative anemia, operation type and primiparity as covariates, while the model for log-transformed length of stay included pre-operative Hb, emergency operation and primiparity rates.

Statistical significance was assessed at the *p* = 0.025 level to account for two outcomes. R statistical software version 4.1.0 (R Foundation for statistical computing, Vienna Austria) was used for all statistical analyses.

## Results

A total of 431 participants were included in the study. During the first two months, 203 women underwent caesarean section delivery and received the usual care, while 228 women delivered during the period where IOCS was mandated for caesarean deliveries.

Before inverse probability weighting, 6/9 pre-partum maternal characteristics were unbalanced with SMD > 0.1 (Table 1). After weighting, the imbalance was removed (all SMD < 0.1).

**Table 1:** Maternal demographic and obstetric data in two cohorts during periods of usual care or where intraoperative cell salvage (IOCS) was mandated for caesarean section deliveries

	Mandated IOCS *n* = 228	Usual Care *n* = 203	Unweighted SMD	Weighted SMD
Age, mean (SD)	31 (5)	30 (6)	0.178	0.001
Gravida, median (IQR)	2.5 (1–3)	2 (1–3)	0.118	0.027
Parity, median (IQR)	1 (0–2)	1 (0–2)	0.004	0.003
Caesarean section, *n* (%)			0.207	0.016
Elective	120 (53%)	86 (42%)		
Emergency	108 (47%)	117 (58%)		
ASA score, *n* (%)			0.101	0.069
1	64 (28%)	57 (28%)		
2	159 (70%)	141 (70%)		
3	5 (2%)	4 (2%)		
4	0 (0%)	1 (1%)		
Anesthesia			0.204	0.006
Spinal	161 (71%)	128 (63%)		
Epidural	42 (18%)	55 (27%)		
Gen. Anesthesia (GA)	14 (6%)	14 (7%)		
CSE	5 (2)	2 (1%)		
Combination^	6 (3%)	4 (2%)		
^Combinations included				
Spinal + GA	2 (1%)	0		
Epidural + GA	4 (2%)	3 (2%)		
Epidural + Spinal + GA	0	1 (1%)		
Pre-operative Hb (g/L), mean (SD)	125 (12)	124 (13)	0.120	0.030
Pre-operative Anemia, *n* (%)	25 (11%)	24 (12%)	0.024	0.075
Pre-operative IV iron, *n* (%)	6 (3%)	22 (11%)	0.068	0.043

There was no difference in allogeneic RBC transfusion, with only one woman in each group receiving blood products. Univariate analysis showed that post-operatively, women in the Mandated IOCS group had higher post-operative Hb values, 111 g/L vs 102 g/L (*p* < 0.001) (despite similar pre-operative Hb, 125 g/L vs 124 g/L), had significantly lower rates of anemia, 48% vs 78% (*p* < 0.001) and less often exposed to post-partum intravenous iron, 11% vs 3% (*p* < 0.001) (Table 2). A higher proportion of women in the Usual Care cohort had an emergency caesarean section (p = 0.03), greater estimated blood loss (EBL) (p = 0.003).

**Table 2:** Post-operative Hb and anemia status of women according to the use of mandated intraoperative cell salvage (IOCS)

	IOCS mandated *n* = 228	Usual care *n* = 203	*p*
Cell salvage deployed, *n* (%)	*n* = 227 196 (86%)	*n* = 203 62 (31%)	< 0.001
Post-operative	*n* = 71	*n* = 95	
Hb (g/L), mean (SD)	111 (14)	102 (12)	< 0.001
Anemia, *n* (%)	14 (20%)	39 (41%)	0.004
Received post-operative IV iron, *n* (%)	6 (3%)	22 (11%)	< 0.001
Estimated blood loss (ml), median (IQR)	400 (300–600)	500 (350–700)	0.003
Blood reinfused, *n* (%)	21 (9%)	6 (3%)	0.008
Reinfusion volume (ml), med (IQR)	*n* = 21 250 (134–338)	*n* = 6 284 (245–290)	0.86

**Fig. 1:**
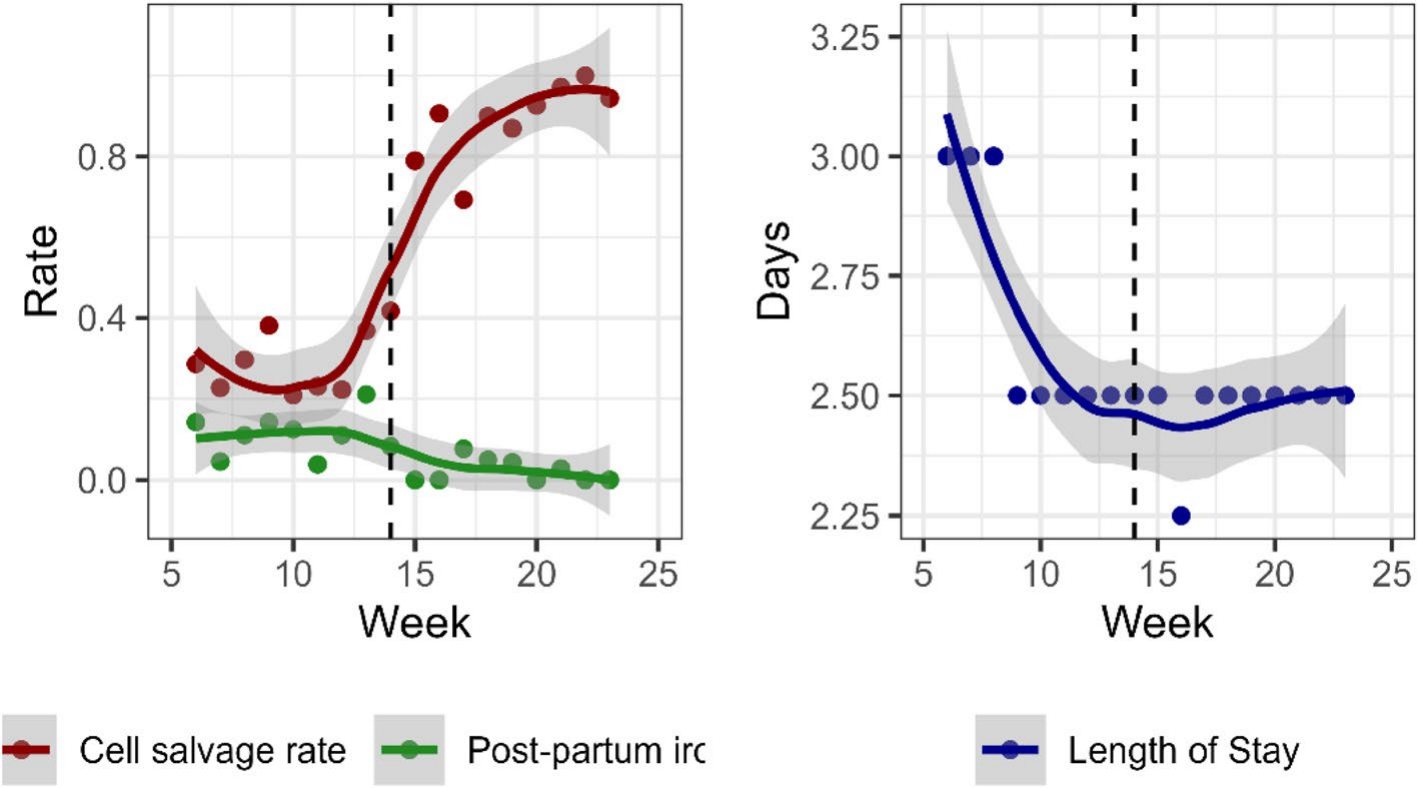
Weekly rates of cell salvage and post-partum IV iron and length of stay in 2020. Lines and shaded confidence bands are produced by lowess smoothing. The dashed vertical line indicates the start of mandated IOCS. TIFF format, resolution 600 dpi, created in Rstudio

Statistical modelling showed that women who were mandated IOCS had lower rates of post-partum iron infusion (odds ratio (OR) = 0.31, 95% CI = 0.12 to 0.80, *P* = 0.016) than women in the Usual Care group (Table 3). After weighting, the length of stay was not different between the groups (geometric mean ratio = 0.93, 95% CI = 0.86 to 1.004, p = 0.063). These results were confirmed by the ITS analysis *(P* = 0.012 and *P* = 0.23 for post-partum iron and length of stay, respectively). Figure 1 shows average weekly cell salvage and post-partum iron rates and length stay before and after mandated IOCS.

**Table 3:** Multivariable statistical modelling with and without inverse probability weighting and interrupted time series (ITS) p-value for a difference between time periods

Outcome	Unweighted Estimate (95% CI)	Unweighted P-value	Weighted estimate (95% CI)	Weighted P-value	ITS *P*-value
Post-partum IV iron^1^	0.22 (0.09–0.56)	0.001	0.31 (0.12–0.80)	0.016	0.012
Length of Stay^2^	0.90 (0.84–0.97)	0.006	0.93 (0.86–1.004)	0.063	0.23

^1^Odds ratio for Yes vs No for mandated intraoperative cell salvage vs Usual Care

^2^Geometric mean ratio for mandated intraoperative cell salvage vs Usual Care

## Discussion

The introduction of routine IOCS for caesarean deliveries during the early phase of the COVID-19 pandemic resulted in higher processing rates and reinfusion volumes of autologous RBC. Consequently, post-partum Hb values were improved and anemia less prevalent in women from this cohort. Intravenous iron prescription occurred less often after delivery, and there was no difference in allogeneic RBC transfusion rates between the two groups. These findings add to the existing evidence demonstrating the safety and efficacy of autologous salvaged blood in the context of LSCS, through the conservation of patient blood and maintained iron stores.

After accounting for the difference between patients at baseline, mandating cell salvage reduced post-operative iron infusion rates. Length of stay, however, appeared to be in decline prior to the COVID restrictions and there was no significant difference between the time periods.

Although not contradicted in this study, IOCS has been shown to reduce the need for allogeneic transfusion of RBC in other surgical settings. [6, 15] In 2012, Liumbruno et al. labelled IOCS “the most practical, effective and useful blood conservation technique in obstetrics” [15]. Due to comprehensive staff education and general enthusiasm for the implementation of PBM among staff across disciplines at our hospital, IOCS was introduced in 2013 for elective and emergency surgery. It quickly became an essential tool for managing maternal bleeding, combined with a specific obstetric hemorrhage bleeding protocol and point-of-care coagulation monitoring. This comprehensive obstetric PBM approach resulted in the lowest RBC transfusion rate in South Australia (personal communication, South Australian Blood, Organ and Tissue Programs). The trial period of mandated IOCS in response to the limited blood availability during the COVID-19 pandemic, demonstrated no further reduction in RBC transfusion rate. It was, however, associated with an improvement in Hb status and reduced rates of post-partum iron deficiency anemia in the Mandated IOCS group compared to those receiving Usual Care. These outcomes may explain the 69% four-month trial period highlight the benefits for the patient-derived from the utilization of IOCS for LSCS.

This study has highlighted the valuable contribution of IOCS in obstetric patient care. The mandated use of IOCS enhanced the effectiveness of an established and successful PBM approach, adding to the pre-existing strong body of evidence. Over the last decade, the outcomes for over 2000 cases of autologous RBC re-infusion after red blood cell recovery during LSCS have been published in the literature. This research has demonstrated the safety, efficacy, and benefits of IOCS, [6, 9–11, 16, 17] regardless of technique, for example differing suction setups, the use or absence of leucocyte depletion filters, and selective versus routine cell salvage provision.

Maternal mortality is increasing in the United States and many parts of the world [10]. Obstetric hemorrhage is the most common cause of maternal death and consequently peri-partum RBC transfusion rates are on the rise, accounting for approximately 3% of all RBC transfusions [18]. It is important to emphasize that these transfusion events occur in young women, who potentially suffer long-term consequences from exposure to allogeneic RBC’s [19, 20]. Bleeding during childbirth can be life-threatening and is notoriously difficult to predict [21]. IOCS in obstetrics is recommended by organizations such as The Association of Anaesthetists of Great Britain and Ireland, The National Institute for Health and Clinical Excellence, The American College of Obstetrics & Gynaecology, The Australian National Blood Authority, the Network for the Advancement of Patient Blood Management, Haemostasis and Thrombosis and the German/Austrian/Swiss Societies of Gynecology and Obstetrics (DGGG, OEGGG, SGGG) [22–27].

However, the availability of this important tool is far from universal and often absent, even in many large obstetric services. In a recent publication, Hofmann et al. screened international efforts and strategies to identify the four main drivers for successful PBM implementation: patient outcomes, cost savings, preventing blood shortages and patient safety [28]. Whether routine IOCS in obstetrics is cost-saving remains unclear, the use of red cell recovery and autotransfusion clearly supports improved patients outcomes, improved patient safety and prevention of blood shortages, all of which have been urgently required during the COVID-19 pandemic [4, 29].

Withholding the provision of IOCS in obstetrics deprives women of the safest form of RBC transfusion, exposing them to preventable anemia and the well-known risks of allogeneic transfusion. Clinicians from around the world caring for women during childbirth should strengthen their efforts to establish IOCS as an integrated modality on a much broader scale [30–32].

### Limitations

The two-month trial of mandated IOCS at our site limited the number of patients included in this study. While we did observe higher rates of re-infusion in the mandated group, a much larger sample size would be required to detect any difference in the donor blood transfusion rates. This sample size would depend on the baseline rate of transfusion, which is already low at our site given our established PBM program. Nonetheless, the benefits of IOCS were still apparent, including lower rates of iron-deficient anemia, a higher post-operative Hb.

In the Usual Care group, cell salvage was initiated for 31% (n = 62/203) of women while 86% of women (n = 196/227) had cell salvage initiated following the mandate. The uptake of cell salvage following the introduction of the mandate started at 48% in week 1, increasing to 93% in week 2. It then remained high throughout the two-month period, ranging from 75 to 96%. Reasons for IOCS not being used include some staff being unaware of new trial clinical protocol and clinician preference.

Interestingly in this study, the EBL was lower in the IOCS Mandate group compared to the Usual Care group. Multiple factors may have contributed to this finding, including the higher number of emergency caesarean sections in the Usual Care IOCS group. In addition, the single-cell salvage set-up may have limited observation of any blood loss, which is already notoriously inaccurate as it relied on visual assessment.

Due to a functional PBM program at our hospital, cell salvage was already occurring in the routine care group, before it was mandated. This represents confounding and may reduce the significance of our observed results. However, it was not the purpose of the study to test cell salvage vs no cell salvage.

The authors acknowledge that while statistically significant results were observed, the clinical significance of this is less certain. We also acknowledge that a study with a significantly larger sample size is required to observe differences in donor transfusion rates and indeed overall cost-effectiveness of mandated IOCS.

## Conclusions

Routine cell salvage provision during LSCS associated with increased post-operative hemoglobin and reduced anemia incidence which may contribute to an avoidance of limited blood products. We also observed a reduction in post-partum iron infusions. Currently, there are a number of barriers to the routine use of IOCS at some sites, including a lack of awareness and funding to support IOCS establishment and protocols for routine IOCS. The improved clinical outcomes we have observed at our site and presented in this manuscript suggest there may be a benefit of using routine intraoperative cell salvage in LSCS. Future research will be conducted to evaluate the economic outcomes associated with increased cell salvage use.

## Data Availability

All data associated with the manuscript and not included in tables, figures or supplemental material are available on request to the corresponding author.
